# Sodium intake assessed by 24-h urine excretion and its relationship with anthropometric measurements in Malaysian adults

**DOI:** 10.1186/s41043-021-00234-1

**Published:** 2021-05-31

**Authors:** Syafinaz Mohd Sallehuddin, Rashidah Ambak, Fatimah Othman, Nur Shahida Abd Aziz, Lalitha Palaniveloo, Noor Safiza Mohd Nor, Rasidah Jamaluddin, Azli Baharudin, Nor Azian Mohd Zaki, Mohamad Hasnan Ahmad

**Affiliations:** 1 Institute for Public Health, National Institutes of Health, Ministry of Health Malaysia, Selangor, Malaysia; 2 Policy and Strategic Planning Section, Allied Health Science Division, Ministry of Health Malaysia, Putrajaya, Malaysia; 3 Sarawak General Hospital, Ministry of Health Malaysia, Kuching, Sarawak Malaysia

**Keywords:** Sodium intake, 24-h urinary sodium excretion, Body mass index (BMI), Waist circumference (WC), Malaysian Community Salt Survey (MyCoSS)

## Abstract

**Background:**

Sodium intake is associated with anthropometric measurement including weight, waist circumference (WC), and body mass index (BMI). Higher intake of sodium is usually linked to higher risk of obesity among adults globally, especially in developing countries. This study aims to explore the probable relationship between sodium intake by 24-h urine excretion assessment and anthropometric measurement of adults in Malaysia.

**Methods:**

A cross-sectional study was conducted from October 2017 to March 2018 using a multi-stage stratified sampling method among Malaysian adults aged 18 years old and above. Sodium intake was determined by 24-h urinary sodium excretion, estimated from the respondents’ 24-h urinary sample. Height was obtained based on standard protocol. Weight and WC were measured twice using validated anthropometric equipment and BMI was calculated according to World Health Organization (WHO) 1998 classification. Descriptive analysis was done to describe socio-demographic characteristics. A simple linear regression and multiple linear regression tests were done to assess the relationship of 24-h urinary excretion and anthropometric measurement. All statistical analysis was done using SPSS version 22.0.

**Results:**

Of 1047 interviewed respondents, 798 respondents had done the 24-h urine collection (76.0% response rate). Majority was between 40 and 59 years old (43.5%) and married (77.7%). Simple linear regression showed a significant positive linear association between 24-h urinary excretion and household income, WC, and obese group. In the multivariate analysis, it was indicated that, an increase of 1 unit of BMI will significantly increase the sodium intake by 129.20 mg/dl and an increase of 1 cm of WC will significantly increase the sodium intake by 376.45 mg/dl.

**Conclusion:**

Our study showed a positive significant relationship between sodium intake estimated by 24-h urinary sodium excretion and BMI of Malaysian adults. More research is suggested on how sodium control can potentially contribute to obesity prevention.

## Background

One of the growing public health concerns worldwide is obesity. This is a state of excessive body fat accumulation [1]. Among other risk factors, studies have found that sodium intake is associated with body weight and increased body mass index (BMI) [2]. Compared to individuals on lower sodium diets, those with a high sodium diet are prone to a 20% increased risk of central obesity [2]. Based on a study carried out in Denmark, it reported an association between body fat mass and sodium intake [3]. Several other local and international studies reported the relationship of increased sodium intake with risk of hypertension, stroke, cardiovascular diseases, and obesity [4–6].

One of the essential nutrients needed by the body is sodium, to regulate normal body physiological function [7]. However, Malaysian Adult Nutrition Survey (MANS) 2003 reported Malaysian sodium intake was 2575 mg/day [8] or 29% higher than the recommendation of the World Health Organization (WHO) that recommends less than 5 g/day of salt intake [9]. Some studies have proposed the relationship between salt and obesity occurs via additional calories consumed and a high sodium intake from a mix of energy-dense processed foods and sugar sweetened beverages [1, 10]. Other findings have suggested that salt may contribute to obesity by increased weight via water retention, genetic influence, salt sensitivity, or altered fat metabolism [2, 9].

In addition, a deprived socio-economic background (SEB) also might have a link to increased risks of obesity and low quality of dietary habits. Those with low SEB may resort to poor quality food choices and eating patterns due to minimal resources. Therefore, they were more likely to consume less nutritious and more calorie-dense food [10]. Earlier international studies mentioned that people with lower SEB, either a lower income or expenses, lower education background, and worked in labor intensive jobs tend to consume a higher intake of meat fat, sugar substitute, and salt; on the other hand, those with better SEB practice good eating practices, control their salt intake level, tend to choose higher grades of food and have more access to healthier food choices including vegetables and fruits [11–13].

Another study reported the relationship of increased sodium intake with risk of obesity and used 24-h urinary excretion as the gold standard method [14]. This study aims to explore the possible relationship between sodium intake estimated from 24-h urinary excretion and anthropometric measurements in Malaysian adults.

## Methods

### Study design

The Malaysian Community Salt Survey (MyCoSS) was a cross-sectional study done nationally from October 2017 to March 2018 using a multi-stage stratified sampling method. A total of 1047 adults aged 18 years and above were included in this study. Face-to-face interviews were the means of data collection for this survey, and it was carried out at the respondents’ homes using tablet mobile devices.

Information on socio-demographic characteristics including age (18 to 39 years old, 40 to 59 years old, and 60 years old and above), sex (male, female), education level (no education, primary, secondary, tertiary), marital status (never married, married and separated/widower), and occupation were obtained.

### Twenty-four-hour urine collection

Respondents’ 24-h urine were collected and measured for sodium 24-h urine excretion. Respondents were instructed to complete a single collection of 24-h urine sample on a day that was convenient for them. Information sheet were given and written consents were obtained from all eligible respondents. The respondents were guided by the researchers on steps of urinary collection both orally and in written form. The first urine was discarded and started from the second urine passed on that day till the first urine of the next were to be collected. The time frame of total collection must be more than 20 h of time intervals. Therefore, they could not miss even 1 time of urine passing otherwise his/her urine collection had to be excluded and asked to repeat the procedure. The respondents were given a reminder tool to ensure they do not miss a urine void. Start and finish time were noted and recorded. Sodium content in the urine was measured using indirect ion-selective electrode method. The definition of an incomplete 24-h urine collection was urinary creatinine < 4 mmol/day for women or < 6 mmol/day for men, 24-h urine volume less than 500 ml for both men and women as well as extreme outliers for urinary creatinine (that is > 3 SDs from the mean) [15]. Conversion from millimoles to grams was made by dividing by a factor of 17 and the conversion from sodium (Na) to salt (NaCl) was by multiplying by a factor of 2.542. The 24-h urinary sodium excretion was then inflated 10% to account for losses in sweat and feces. Sodium intake by 24-h urinary excretion was then compared to the Malaysian dietary sodium of 2000 mg/day (or 5 gm salt or 1 tsp.).

### Anthropometric measurements

The measurements were done by trained researchers according to standard protocol of the World Health Organization (WHO) 1998. Weight was measured with a digital weighing scale (TANITA HD-319) with an accuracy of 0.1 kg, height was measured with SECA 213 stadiometer to the nearest 0.1 cm, and waist circumference (WC) was measured using SECA measuring tape (SECA, Germany) to the nearest 0.1 cm. All the measurements were measured two times using validated and calibrated anthropometric equipment and the average was recorded as the final reading. Body mass index (BMI) was calculated as the ratio of weight in kilograms to the square of height in meters (kg/m^2^) and categorized based on the WHO 1998 guidelines [16].

### Statistical analysis

The characteristics of respondents included sex, age groups, marital status, education level, occupation, and anthropometric measurements were presented in prevalence of percentages. Simple linear regression analysis was done to examine the association between 24-h urinary excretion and changes in BMI and anthropometric measurements. Independent variables which were not significant were excluded in the multiple linear regression model. Interaction and multicollinearity were tested; there were no interaction between independent variable. The test applied was stepwise method and followed by the enter method for confirmation. The best model was chosen according to adjusted regression coefficient value to predict the value of dependent factors. For statistical testing, *p* values ≤ 0.05 were considered statistically significant. The simple and multiple linear regression analysis was done using IBM SPSS Statistics for Windows, version 22.0.

## Results

From the 1047 respondents that were interviewed, about 798 respondents had done the 24-h urine collection (76.0% response rate).

The characteristics of the study respondents are shown in Table 1. Most of the respondents had up to secondary school education (51.1%), while 18.2% only studied up to primary school and about 5.1% did not receive any formal education. The remaining respondents received tertiary education (25.6%). By age groups, almost half of the respondents were between age 40 to 59 years old (43.5%). The majority of respondents was married (77.7%) and most were housewives (23.5%), followed by others. Almost half of the respondents were earning within RM 1000.00 to RM 3999.00 (47.0%).

**Table 1:** Socio-demography characteristic of respondents (*n* = 798)

Socio-demographic characteristics	***n***	Percentage
**Sex**		
Men	340	51.7
Women	458	48.3
**Age groups**		
18–39	235	29.4
40–59	347	43.5
60+	216	27.1
**Marital status**		
Never married	93	11.5
Married	594	77.7
Separated/widower	110	2.5
**Education level**		
None	64	5.1
Primary	167	18.2
Secondary	383	51.1
Tertiary	184	25.6
**Occupation**		
Public sector	117	14.0
Private sector	126	17.2
Self-employed	179	23.0
Housewives	214	23.5
Unemployed	162	22.3
**Household Income**		
< 1000	237	29.8
1000–3999	374	47.0
> 4000	185	23.2
**Anthropometry**		
**Body mass index (BMI)**		
Underweight	35	4.5
Normal	285	35.2
Overweight	287	37.5
Obese	191	22.8
**Waist circumference**		
Male		
< 90	410	48.7
≥ 90	387	51.3
Female		
< 80	180	20.1
≥ 80	618	79.9

Prevalence by BMI categories for underweight was 4.5%, followed by normal weight with 35.2%, overweight with 37.5% and for obese was 22.8%. As for male, the prevalence for waist circumference above 90 cm was 51.3% and for female prevalence of waist circumference above 80 cm, was 79.9%.

In the simple linear analysis shown in Table 2, independent variables with *p* < 0.05 were included in the multiple linear regression model. Simple linear regression analysis showed significant positive linear associations between sodium intake assessed by 24-h urine excretion with household income, waist circumference, and obesity. Results also showed a significant negative linear association between sodium intake assessed by 24-h urine excretion with sex, age group, marital status, occupation, and being underweight.

**Table 2:** Predictive factors of sodium intake measured by 24-h urinary excretion based on simple linear regression analysis

Characteristics	*b*^*a*^ (95% CI)	*p* value*
Sex		
Male^r^	0.0 (0.00, 0.00)	0.000
Female	− 598.01 (− 791.85, − 404.17)	0.001*
Age		
18–39^r^	0.0 (0.00, 0.00)	0.000
40–59	− 20.70 (− 218.47, 177.05)	0.837
60+	− 232.88 (− 453.08, − 12.67)	0.038*
Marital Status		
Never married^r^	0.0 (0.00, 0.00)	0.000
Married	209.55 (− 15.26, 434.36)	0.068
Separated/widower	− 363.71 (− 504.59, − 222.83)	0.001*
Education level		
None^r^	0.00 (0.00, 0.00)	0.000
Primary	− 98.10 (− 338.78, 142.58)	0.424
Secondary	82.61 (− 113.53, 278.76)	0.409
Tertiary	223.83 (− 8.204, 455.85)	0.059
Occupation		
Public sector^r^	0.0 (0.00, 0.00)	0.000
Private sector	181.21 (− 87.07, 449.50)	0.185
Self-employed	107.27 (− 10.13, 224.67)	0.073
Housewives	− 115.09 (− 188.36, − 41.82)	0.002*
Unemployed	− 60.24 (− 130.60, 10.11)	0.093
Household income		
< 1000^r^	0.0 (0.00, 0.00)	0.000
1000–3999	76.86 (− 119.50, 273.22)	0.443
> 4000	253.48 (22.03, 484.92)	0.032*
Waist circumference (cm)		
Men		
< 90^r^	0.0 (0.00, 0.00)	0.000
≥ 90	769.64 (451.40, 1087.87)	0.001*
Women		
< 80^r^	0.0 (0.00, 0.00)	0.000
≥ 80	444.53 (181.10, 707.95)	0.001*
BMI		
Normal^r^	0.00 (0.00, 0.00)	0.000
Underweight	− 855.54 (− 1329.98, − 381.10)	0.001*
Overweight	51.79 (− 50.22, 153.82)	0.319
Obese	186.56 (111.15, 261.97)	0.001*

*Significant at *p* value at *p* < 0.05

^r^Reference factor in dummy variable

Dependent variable: sodium intake by 24-h sodium excretion

*b*^*a*^ unadjusted regression coefficient

However, in multivariate analysis, which is shown in Table 3, age, sex, BMI, waist circumference, and marital status were significantly correlated with sodium intake measured by 24-h urine excretion.

**Table 3:** Predictive factor of sodium intake measured by 24-h urinary excretion based on multiple linear regression (multivariable analysis)

Characteristics	*b*^*a*^ (95% CI)	*p* value*
Sex		
Female	− 563.80 (− 761.50, − 366.11)	0.001*
Age		
60+	− 228.45 (− 443.58, − 13.31)	0.037*
Marital status		
Separated/widower	− 251.03 (− 393.28, − 108.77)	0.001*
Waist circumference (cm)		
Men ≥ 90	376.45 (158.60, 594.30)	0.001*
BMI		
Underweight	− 638.76 (− 1097.93, − 179.58)	0.006*
Obese	129.20 (45.13, 213.27)	0.003*

*Significant at *p* value at *p* < 0.05

Dependent variable: sodium intake by 24-h sodium excretion

*b*^*a*^ adjusted regression coefficient

Findings indicate that an increase of 1 unit of BMI will significantly increase the sodium intake by 129.20 mg/dl whereas for waist circumference, increase in 1 cm of waist circumference will increase significantly the sodium intake by 376.45 mg/dl.

## Discussion

This study was the first to assess the association between sodium intake with anthropometric measurements among adults in Malaysia. The findings showed that overweight individuals had the highest sodium intake. This is likely a result of diet and lifestyle habits practiced by Malaysians which include high-calorie intake from calorie-dense food, processed food, sugar sweetened beverages, or just plain excess calorie intake [15]. The finding also showed that both males and females had an average larger waist circumference than the recommendation. This could be attributed to excess calorie intake causing thirst and leading to increased fluid intake including sugary drinks, which then contribute to increased energy intake. This finding is supported by few studies in other countries suggesting evidence of a direct association between sodium intake and obesity [6, 15].

These findings also found positive significant associations between sodium intake by 24-h urinary excretion with lower household income, larger waist circumferences, and obesity. For such individuals, their lower income possibly limits their food choices to cheaper processed food that are high in fat content and calories. This ties in with the World Health Organization’s (WHO) statements on socio-economic factors influencing health where “a higher income and social status are associated with better health” and “low education background is associated with poor health” [17]. The findings were also supported by a Japanese study which found that working class respondents had higher urinary sodium excretion thereby indicating higher salt intake [12]. Other studies reported that urinary sodium excretion was significantly higher in overweight or obese individuals than those with normal weight, which was in line with the current findings which suggest salt intake does affect weight status [18, 19].

Higher energy intake also results in an increased risk of higher salt intake as mentioned above. From the regression analysis, there was a positive relationship between urinary sodium excretions with weight (Table 3), where increased sodium intake is associated with increased body weight. This association was found to be stronger in men, possibly due to males having larger body frames in general and therefore tend to have higher calories intake [19, 20].

The finding is similar to another Asian studies that described the positive association between sodium intake and body weight could be caused by high intake of high-calorie, sugary drinks and high-salt food [21].

To our knowledge, this is the first population-based study examining salt intake of adults in Malaysia. It utilized the 24-h urinary sodium excretion method which is considered the gold standard for assessing sodium intake. However, limitations include not taking into account food intake by 24-h dietary record and food diary.

## Conclusion

There was a significant positive relationship between sodium intake that was measured by 24-h urinary sodium excretion and BMI of adults in Malaysia. This emphasizes that too much sodium intake may lead to obesity-related health problems. Further researches are necessary to explore the possible outcome of increased sodium intake with obesity effect.

## Data Availability

The datasets used and/or analyzed during the current study are available from the corresponding author on reasonable requests.

## Abbreviations

MyCoSS: Malaysian Community Salt Survey
BMI: Body mass index
WC: Waist circumference
WHO: World Health Organization
